# Transplanting human infant gut microbiome species into *Galleria mellonella*

**DOI:** 10.1186/s13104-024-06785-w

**Published:** 2024-04-30

**Authors:** Harriet C.C. Gooch, Marjorie Labedan, Lindsay J. Hall, Anthony Maxwell

**Affiliations:** 1 Department of Biochemistry and Metabolism, John Innes Centre, Norwich Research Park, Norwich, NR4 7UH UK; 2 Quadram Institute Bioscience, Norwich Research Park, Norwich, NR4 7UQ UK; 3 Department of Molecular Microbiology, John Innes Centre, Norwich Research Park, Norwich, NR4 7UH UK; 4 Present address: Department of Ecology and Evolution, University of Lausanne, Lausanne, 1015 Switzerland; 5 Present address: Institute of Microbiology and Infection, University of Birmingham, Birmingham, B15 2TT UK

**Keywords:** *Galleria*, Microbiome, *Enterococcus*, *Bifidobacterium*

## Abstract

**Objective:**

Study of the human infant gut microbiome requires the use of surrogate mammalian species such as mice. We sought to investigate the usefulness of the greater wax moth larva, *Galleria mellonella*, as an alternative.

**Results:**

We have analysed the native gut microbiome of Galleria and developed methods for clearing the native microbiome and introducing species from human infant faecal samples. We find that some species, e.g. enterococci, are more successful at recolonisation, but that others, e.g. *Bifidobacterium*, are less so. The work paves the way for using *Galleria* rather than mice in this and similar work.

**Supplementary Information:**

The online version contains supplementary material available at 10.1186/s13104-024-06785-w.

## Introduction

The greater wax moth, *Galleria mellonella*, is a member of the order Lepidoptera and is mostly known as a pest of beehives [1]. As *G. mellonella* can grow for several generations on artificial food [2], are easily inoculated with bacteria, can grow at 37 °C, and have few ethical issues, it has gained in popularity as a model host, in particular to study microbial interactions such as pathogenesis [3, 4]. Previously, most studies have used larvae supplied from pet food shops, which may have been grown with antibiotics and hormones, but recently there have been attempts to standardise *G. mellonella* as a model [5, 6], including TruLarv™ [7].

The bacterial population in the *G. mellonella* gut is low in both diversity and abundance. Most studies report the gut microbiome to be primarily composed of *Enterococcus*. The relationship *G. mellonella* has with its commensal enterococci may make it a useful model to study *Enterococcus* commensal bacteria from humans.

Here we aimed to expand the use of *G. mellonella* beyond infection studies and assess its potential as a surrogate for studying the gut microbiome of human infants. Given the microbial community in human early life is relatively low diversity and is often dominated by beneficial genera such as *Bifidobacterium* [8], we sought to determine if *G. mellonella* could prove a useful model for understanding dynamics in these burgeoning ecosystems, which could also be used to test the utility of different probiotic formulations.

## Methods

*Insect rearing: Galleria mellonella* larvae were obtained from a colony originally sourced from Livefood UK Ltd and maintained at the John Innes Centre Entomology Facility (Norwich, UK). Where specified, larvae were from BioSystems Technology (TruLarv™), and also from a local Norfolk beekeeper. Larval diet consisted of: 20 g brown sugar (Sainsbury’s dark soft brown sugar), 40 mL glycerol (Sigma), 20 g milk powder (Dried Skimmed Milk Powder, Marvel), 20 g wholemeal flour (Strong Stoneground 100% Wholemeal Flour, Sainsbury’s), 10 g yeast extract (Merck) 10 g wheat germ (Neal’s Yard Wholefoods Natural Wheatgerm), 40 g bran (Neal’s Yard Wholefoods Natural Wheat Bran). Unless specified, larvae were incubated at 30 °C.

### Dissection and DNA isolation

Larvae were transferred to empty 90 mm round Petri dishes for 2 h of starvation; dissection was carried out under sterile conditions [9]. Larvae were surface-sterilised by washing in 70% ethanol followed by sterile distilled water, transferred individually to small centrifuge tubes and killed by flash-freezing in liquid nitrogen, then the head was removed and a cut was made down the ventral side. Gut contents were removed with forceps and placed in a sterile 2 mL tube, three guts to a tube. Both gut and whole larval samples were homogenised using an MP Biomedicals FastPrep-24™ homogeniser with 2 mL Lysing Matrix D tubes, for 40 s (speed 6.0). DNA was purified with the FastDNA® SPIN Kit for Soil according to instructions but with two extra homogenisation cycles followed by 15-min centrifugation.

*Characterisation of the native G. mellonella gut microbiome*: Library preparation for 16 S rRNA gene amplicon sequencing of whole *Galleria* guts was carried out according to Illumina [10]; the following protocol was used: a cycle of 94 °C 3 min and 25 cycles of 94 °C for 45 s, 55 °C for 15 s and 72 °C for 30 s. V3-V4 amplicon sequencing was carried out by Novogene using the Novoseq 6000 PE150 platform. Taxa were assigned using Centrifuge (v0.15). Primers: 341 F: CCTAYGGGRBGCASCAG; 806R: GGACTACNNGGGTATCTAAT.

*Clearing the native G. mellonella gut microbiome*: Larvae were fed on food containing 15 mg streptomycin (Sigma) and 15 mg oxytetracycline (Sigma) per 100 g of food for 0, 1, 5 or 10 days. Larvae were dissected, guts removed, homogenised and plated. Full-length 16 S rRNA amplification through PCR was carried out on gut homogenates and products run on 1% agarose gels and stained with ethidium bromide. In subsequent experiments, larvae were fed as above for 10 days, then incubated for two days without food. Primers: 8 F: AGAGTTTGATCATGGCTCAG [11]; 1492R: TACGGTTACCTTGTTACGACT [12].

*Faecal slurry stock*: Faecal collection from the Norfolk and Norwich University Hospital NNUH was approved by the Faculty of Medical and Health Sciences Ethics Committee at the University of East Anglia (UEA), and followed protocols laid out by the UEA Biorepository (License no: 11,208) [13]; material was frozen at -80 °C within 24 h, a procedure known to minimise species loss [13]. A faecal slurry stock was made using 30 unique 1 g samples from different infants. Under sterile, anaerobic conditions the samples were combined into a single 50 mL Falcon tube, and 20 mL of sterile PBS (Formedium) was added. The slurry was then vortexed until homogenous. 1 mL aliquots were flash-frozen in liquid nitrogen, and stored in cryo-vials at -80 °C.

### Gut colonisation

A 1 g aliquot of human infant faecal slurry was mixed into 10 g of sterile food in each of 3 Petri dishes. 12 larvae were placed on food then incubated at 37 °C. On day 0, guts were dissected from 9 antibiotic-treated larvae, followed by 9 larvae each from the control and faecal slurry groups on days 1, 2, 4 and 8. Guts were pooled, 3 per sample, and homogenised using glass beads in an Omni Bead Ruptor (Speed 4 for 2 × 60 s, 30 s dwell time). Samples were diluted 1000x in PBS, spread on BHI plates and incubated for 24 h at 37 °C.

### Mortality assessment

Mortality was assessed by stimulating larvae and monitoring for movement. Health was assessed using the health index [14].

## Results

**Fig. 1:**
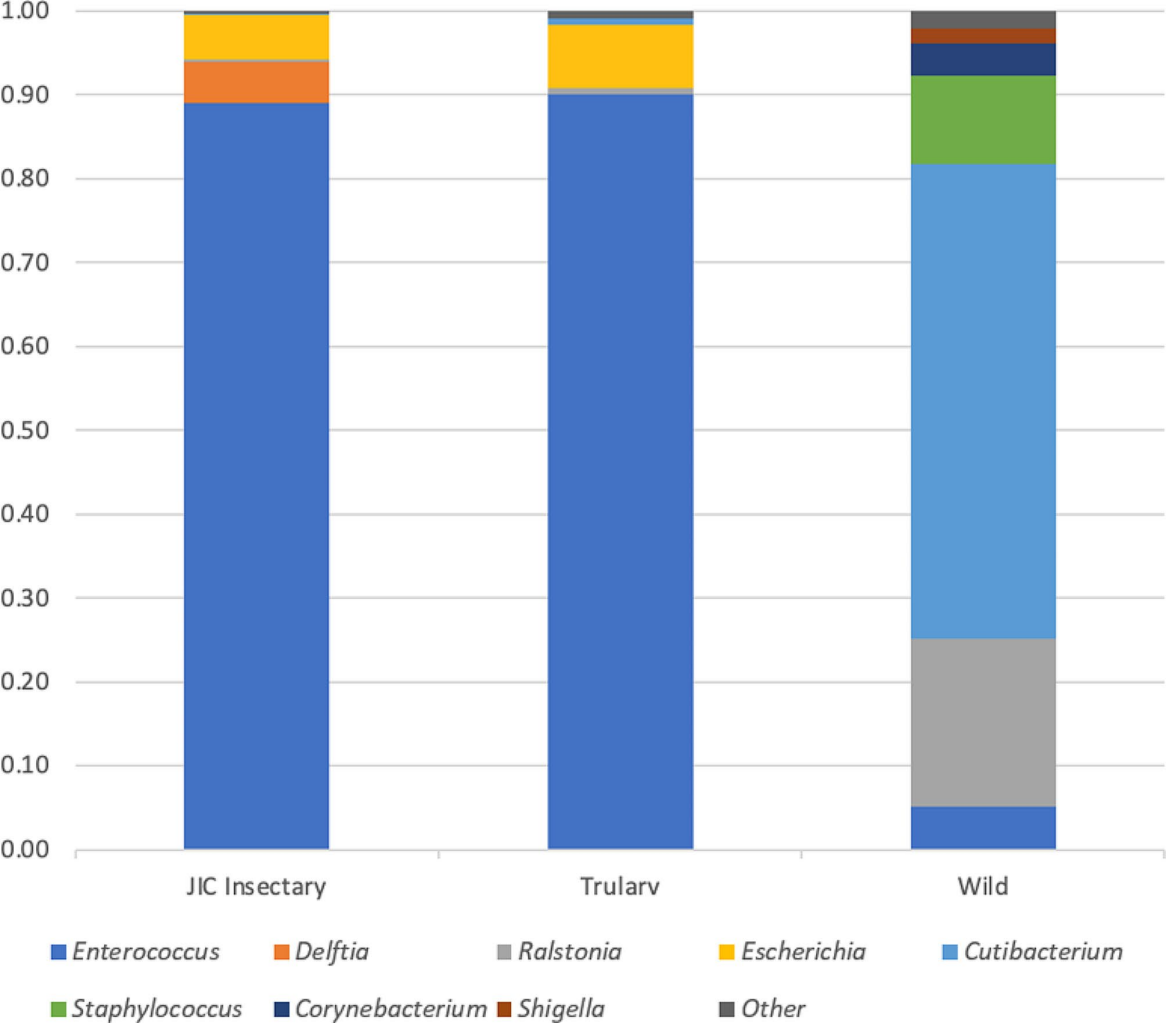
Composition of the gut microbial communities of *G. mellonella* larvae from different sources, by 16 S rRNA amplicon sequencing. Larvae were flash-frozen, guts dissected and pooled 3 to a sample; DNA was purified and sequenced using 16 S rRNA gene amplicon sequencing (V3-V4). Reads were classified using Centrifuge v0.15 [17]. Relative abundances < 0.5% grouped under ‘Other’

*Native gut microbiome of G. mellonella*: Initially we investigated the native microbiome of our experimental colonies: in-house, TruLarv™ (a commercial source of research-grade larvae), and a wild colony. Flash-frozen whole larvae and dissected larval guts, homogenised using a bead beater, were serially diluted in PBS, plated on BHI medium and incubated for 24 h. Cell counts were 3.98 × 10^6^ cfu per whole larva and in guts 2.36 × 10^5^ cfu per larva. Each sample contained purified DNA from three pooled *G. mellonella* gut contents. 16 S rRNA genus level taxonomic profiles (Fig. 1) for the larvae from our colony and the Trularv™ larvae were very similar and were dominated by *Enterococcus.* as usually reported [15]. During the course of this analysis a new bacterial species, *Enterococcus innesii*, was isolated and is reported elsewhere [9]. The composition of the gut microbiome of the wild larvae appeared very different; it was dominated by *Cutibacterium* and also contained *Ralstonia* and *Staphylococcus*. These bacteria are common members of the ‘kitome’ [16] and may have arisen due to contamination. Very few bacteria could be isolated from the wild larvae, so the bacterial abundance in the guts may have been very low. These larvae were melanised (i.e. encapsulated with melanin) before sampling so may have had high immune activity, which may disrupt the gut microbiome.

### Clearing the G. mellonella gut of bacteria

We developed a protocol to clear the *Galleria* gut of its native bacteria by feeding with oxytetracycline and streptomycin. Guts were dissected out, homogenised, and DNA purified. Prior to DNA purification, the homogenate was plated at 1/100 dilution on BHI plates. No colonies grew from the 5- or 10-day gut samples. Fig. S1 shows PCR products of 16 S rRNA amplification of the purified DNA. A band can be seen for the 1-day and 5-day samples but not the 10-day, showing that a 10-day antibiotic treatment is preferable.

### Limited colonisation of the Galleria gut can be achieved with faecal slurry

Larvae were placed on food containing faecal slurry and incubated for 1, 2, 4 and 8 days, alongside a control fed only sterile food. No colonies were apparent for larvae fed sterile food and many identical-looking colonies were observed for larvae fed faecal slurry. Using 16 S rRNA PCR we identified these isolates as *E. faecalis.* Due to the poor resolution of 16 S rRNA gene amplicon sequencing for differentiating at the species level, it is possible that this is a resurgence of the host *Enterococcus*, but we consider this unlikely due to the absence of cultures on the control plates.

### Faecal slurry colonisation persists over several days

**Fig. 2:**
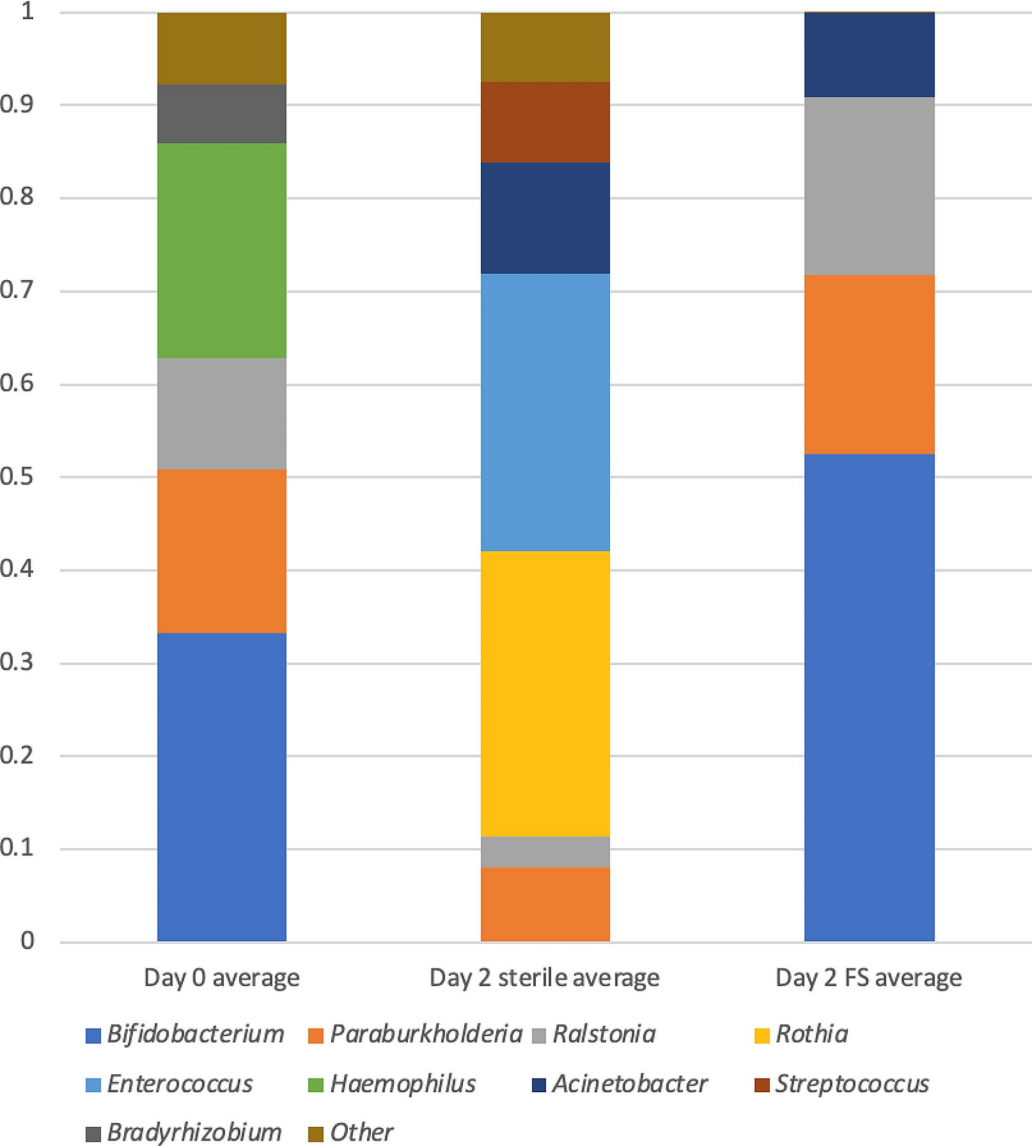
Gut composition by genus following faecal slurry feeding. Antibiotic-treated larvae were fed faecal slurry (FS) for 2 days before they were flash frozen and homogenised. DNA was purified and subjected to 16 S rRNA gene amplicon sequencing (V3-V4). Taxa were assigned using Centrifuge (v0.15)

**Fig. 3:**
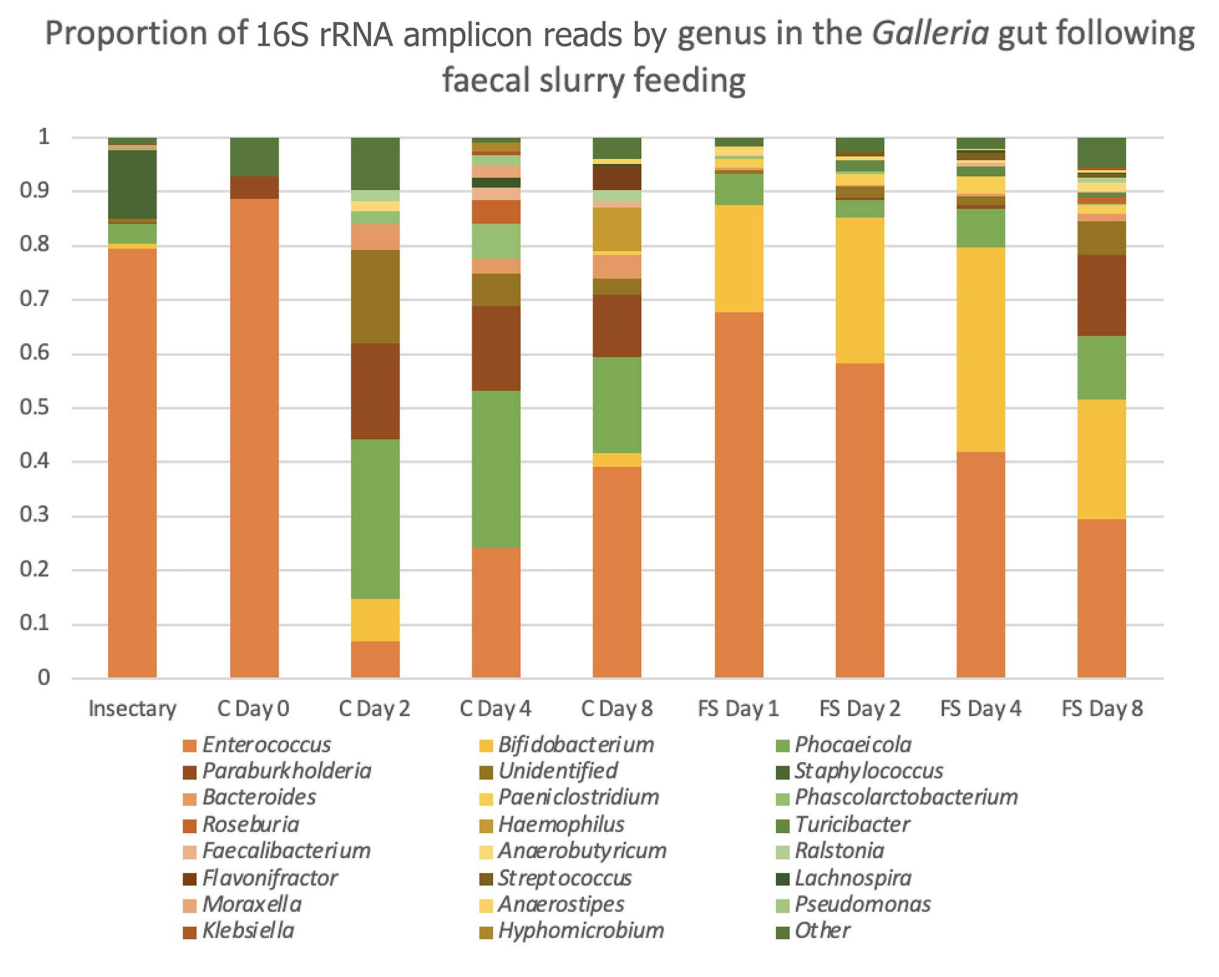
Gut composition by genus following faecal slurry feeding. Antibiotic-treated larvae were fed faecal slurry (FS) or sterile food (C) for up to 8 days, frozen and analysed as in Fig. 2

Larvae fed faecal slurry have a higher proportion of *Bifidobacterium* than both the larvae sampled before the experiment and the larvae not fed faecal slurry (Fig. 2). The resulting bacterial composition over longer times can be seen in Fig. 3. The faecal-slurry-fed larvae had a higher abundance of *Bifidobacterium*, which is very abundant in the infant gut microbiome, and is similar to what was observed in the clinical study from where these samples were sourced [8]. However, both faecal-slurry-fed larvae and control larvae converge to similar profiles after 8 days. In most of the groups, the most dominant genus remains *Enterococcus*, perhaps reflecting its dominance in the native *Galleria* gut.

## Conclusions/discussion

We aimed to investigate the possibility of colonising *G. mellonella* larvae with bacteria from human infant gut. This required a number of steps. Investigation of the native gut microbiome of *G. mellonella* larvae showed that cultured larvae from two different sources contained similar bacterial species, but bacteria from larvae from a ‘wild’ source were potentially quite different, emphasising the importance of using well-characterised sources of larvae. Prior to the attempted colonisation with non-native species, it was necessary to clear the larvae of their resident gut microbiome. We have established a protocol for the treatment of *G. mellonella* to efficiently clear the microbiome using only oxytetracycline and streptomycin. Following this treatment, a break of 2–3 days must be given to ensure the absence of antibiotic when attempting to colonise larvae.

Overall it is unclear how successful colonisation of the *G. mellonella* gut with infant gut bacteria was. Bifidobacteria are the main taxon in the early infant gut [8] so the high proportion of *Bifidobacterium* seen in the larval gut following feeding with faecal slurry is promising. However, 16 S rRNA amplification doesn’t discriminate between living and dead bacteria, so it is unclear whether they were viable. Furthermore, given the more aerobic environment in the larvae gut (i.e. higher oxygen levels [18]), this may not represent an optimal niche for longer-term colonisation of anaerobic *Bifidobacterium* species and strains.

The high proportion of *Enterococcus* in the faecal-slurry-fed larvae is unsurprising given it is dominant in the native *G. mellonella* microbiome [15, 19]. Our recent work indicates that a newly identified novel *G. mellonella*-resident *Enterococcus* species (*E. innesii*), is also closely related to strains that have been isolated from human patients [9]. However, the composition of the faecal-slurry-fed microbiome is not stable; whether or not the infant gut bacteria are viable in the gut, they do not seem to be able to permanently colonise the gut. The composition eventually converges to a similar profile as the other antibiotic-treated larvae (Fig. 3), which may link to the differences in gut physiology between humans and insects. The peritrophic membrane, which encapsulates the food bolus in insects, prevents bacteria having proximity to the epithelial cells and therefore excludes the human commensals from the mucosal niches they would inhabit in a mammalian gut. The conditions in the lepidopteran gut also differ from the conditions in the mammalian gut in other ways, such as oxygen levels and pH, which is much more alkaline than any part of the human gut [20]. Although there is plenty of fibre in the diet, the lack of access to other sources of nutrition, such as mucus or human milk oligosaccharides from breast milk, may also be limiting growth of the infant-gut bacteria. Mortality of the larvae fed faecal slurry was low, however, they did not survive pupation, as with all larvae that were antibiotic-treated. This may be due to the absence of bacteria providing colonisation resistance [19]. Taken together our results demonstrate a viable protocol for introducing non-native (human infant) bacteria into *G. mellonella* larvae.

### Limitations

We found that, although some species (e.g. enterococci) may, at least temporarily, thrive in the insect gut, others (e.g. bifidobacteria) may not colonise longer-term. So, although the success of this endeavour was limited. it does provide the methodology to explore this approach further, e.g. utilising deeper microbiota profiling approaches such as shotgun metagenomics, and also other human microbiota-associated samples from adults and different disease states, e.g. enteric infections.

### Electronic supplementary material

Below is the link to the electronic supplementary material.

Supplementary Material 1

## Data Availability

All data/materials can be obtained by contacting the senior author (tony.maxwell@jic.ac.uk). Sequencing data have been submitted to European Nucleotide Archive (ENA) database, accession number: ERP157621 (https://www.ebi.ac.uk/ena/browser/view/PRJEB72824).

## Abbreviations

FS: Faecal slurry
HMO: Human milk oligosaccharide
PCR: Polymerase chain reaction
UEA: University of East Anglia
