# Supplementary material for: Transplanting human infant gut microbiome species into Galleria mellonella
Source: BMC Res Notes. 2024 Apr 30;17:123. doi: 10.1186/s13104-024-06785-w (PMC11060968; doi:10.1186/s13104-024-06785-w)
Supplement: Supplementary file 1 — Supplementary Material 1 [file 13104_2024_6785_MOESM1_ESM.docx]

# Supplementary materials

# Transplanting human infant gut microbiome species into *Galleria mellonella*.

Harriet C.C. Gooch^1^, Marjorie Labedan^1,†^, Lindsay J. Hall^2,‡^, Anthony Maxwell^1,3*^

^1^Department of Biochemistry and Metabolism, John Innes Centre, Norwich Research Park, Norwich NR4 7UH, UK

^2^Quadram Institute Bioscience, Norwich Research Park, Norwich, NR4 7UQ, UK

^1^Department of Molecular Microbiology, John Innes Centre, Norwich Research Park, Norwich NR4 7UH, UK

^†^Present address: University of Lausanne, Department of Ecology and Evolution, 1015 Lausanne, Switzerland

^‡^Present address: Institute of Microbiology and Infection, University of Birmingham, B15 2TT, UK

*Correspondence: [tony.maxwell@jic.ac.uk](mailto:tony.maxwell@jic.ac.uk)

A B

| 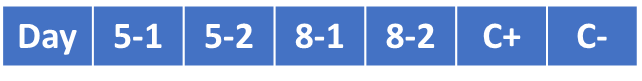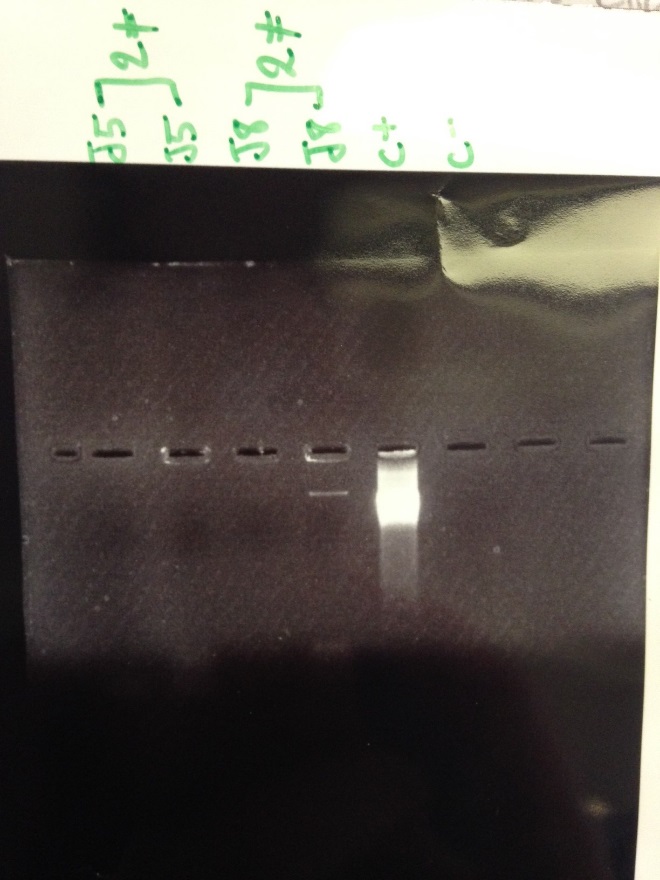  1484 bp 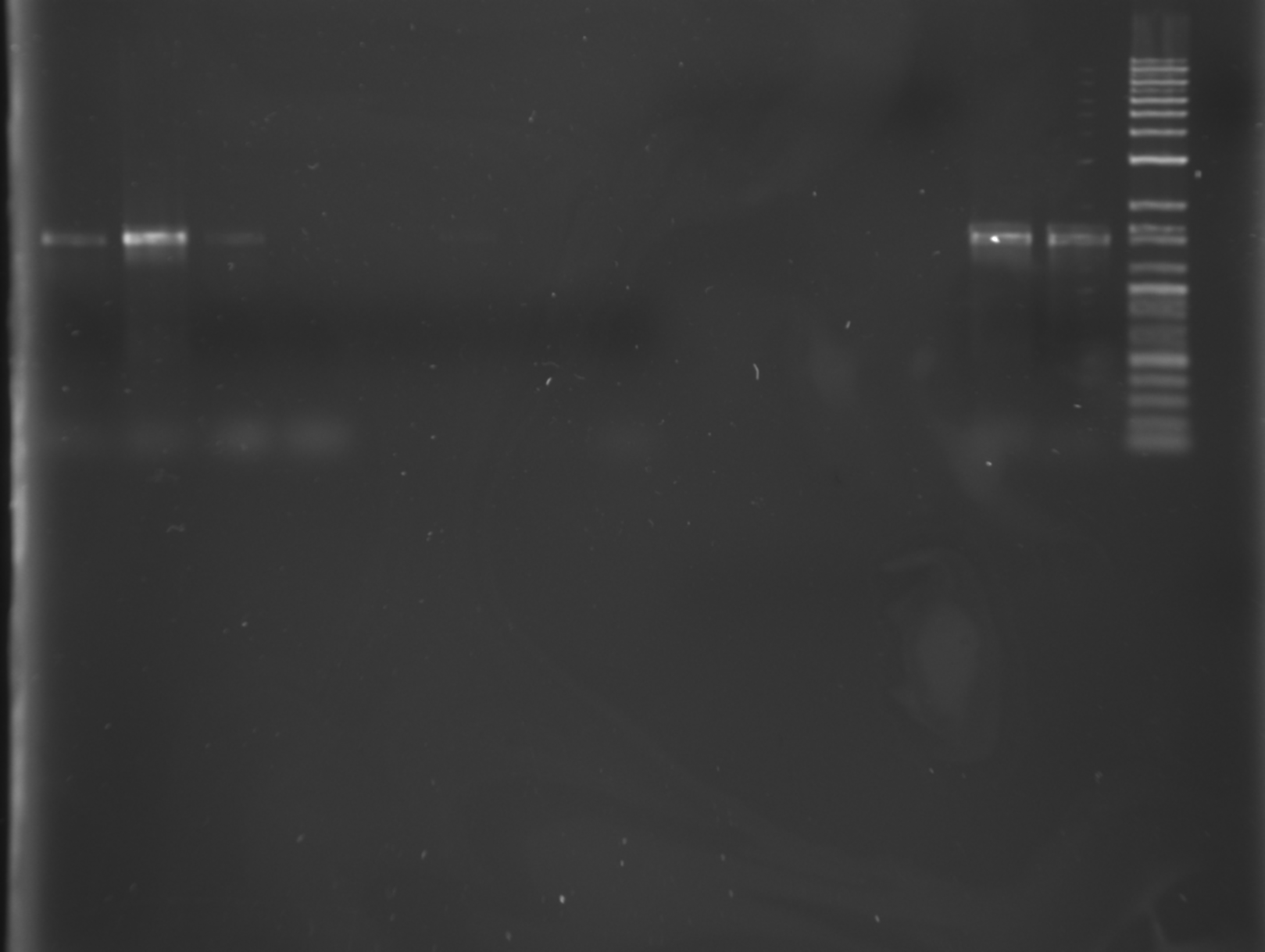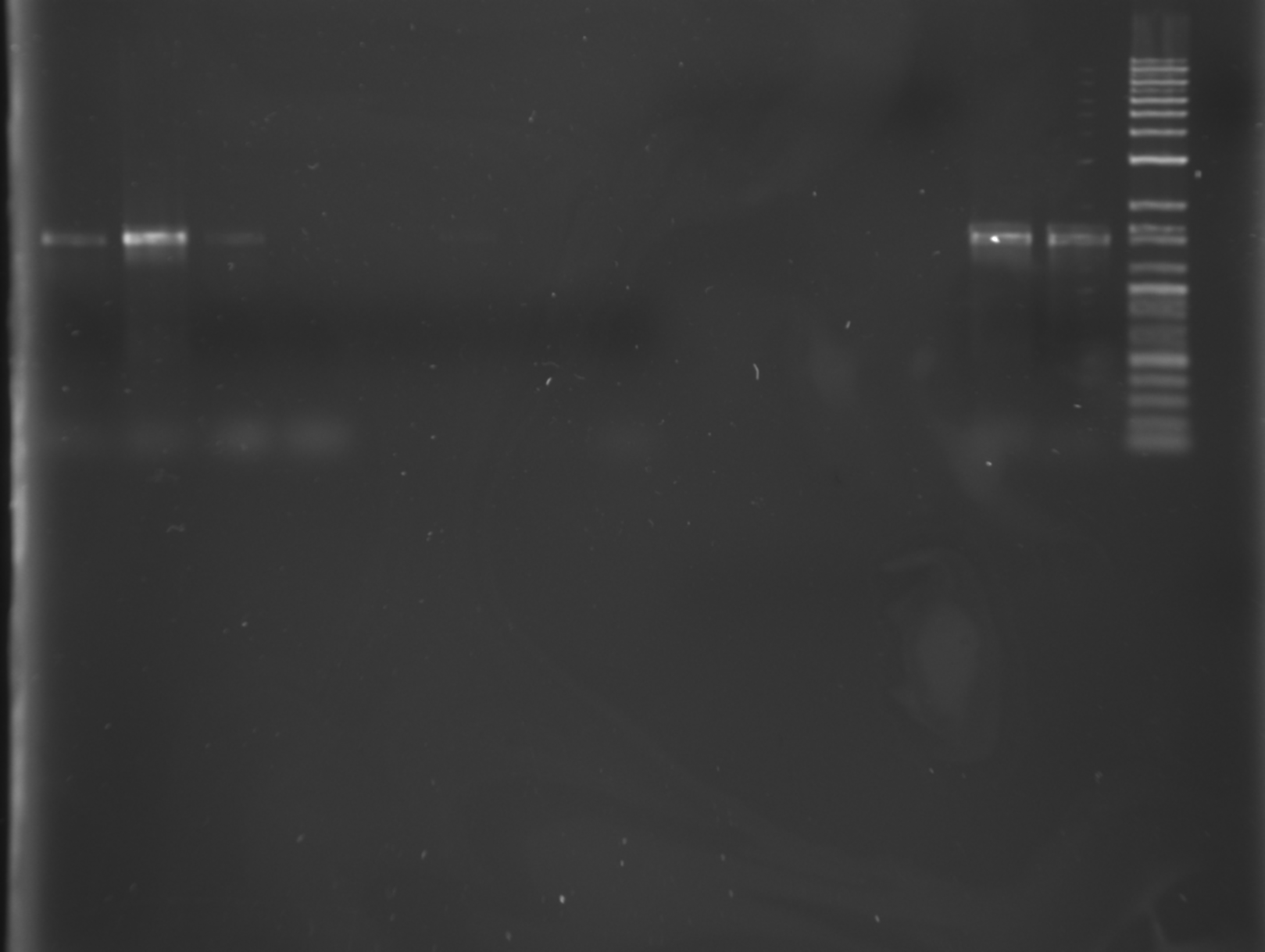 1 day  5 days  No treatment  10 days |
| --- |
| \| **Figure S1: 16S rRNA PCR of purified DNA from gut extracts following antibiotic treatment**. A. Larvae were fed with food containing streptomycin and oxytetracycline (15 mg of each per 100 g food) for 0, 1, 5 or 10 days before being killed by flash freezing. Guts were dissected out and homogenised using bead-bashing. DNA was purified and 16S PCR carried out. B. Shows a similar experiment with duplicate samples taken at 5 and 8 days. C+ indicates a positive control (no antibiotic treatment); C- Indicates a negative control (buffer only). The PCR products are shown here using 1% agarose gel electrophoresis. \| \| --- \| |
